# Supplementary figures and images for: Mitogenomics and phylogenetics of twelve species of African Saturniidae (Lepidoptera)
Source: PeerJ. 2022 Apr 18;10:e13275. doi: 10.7717/peerj.13275 (PMC9022641; doi:10.7717/peerj.13275)

## Slide 1
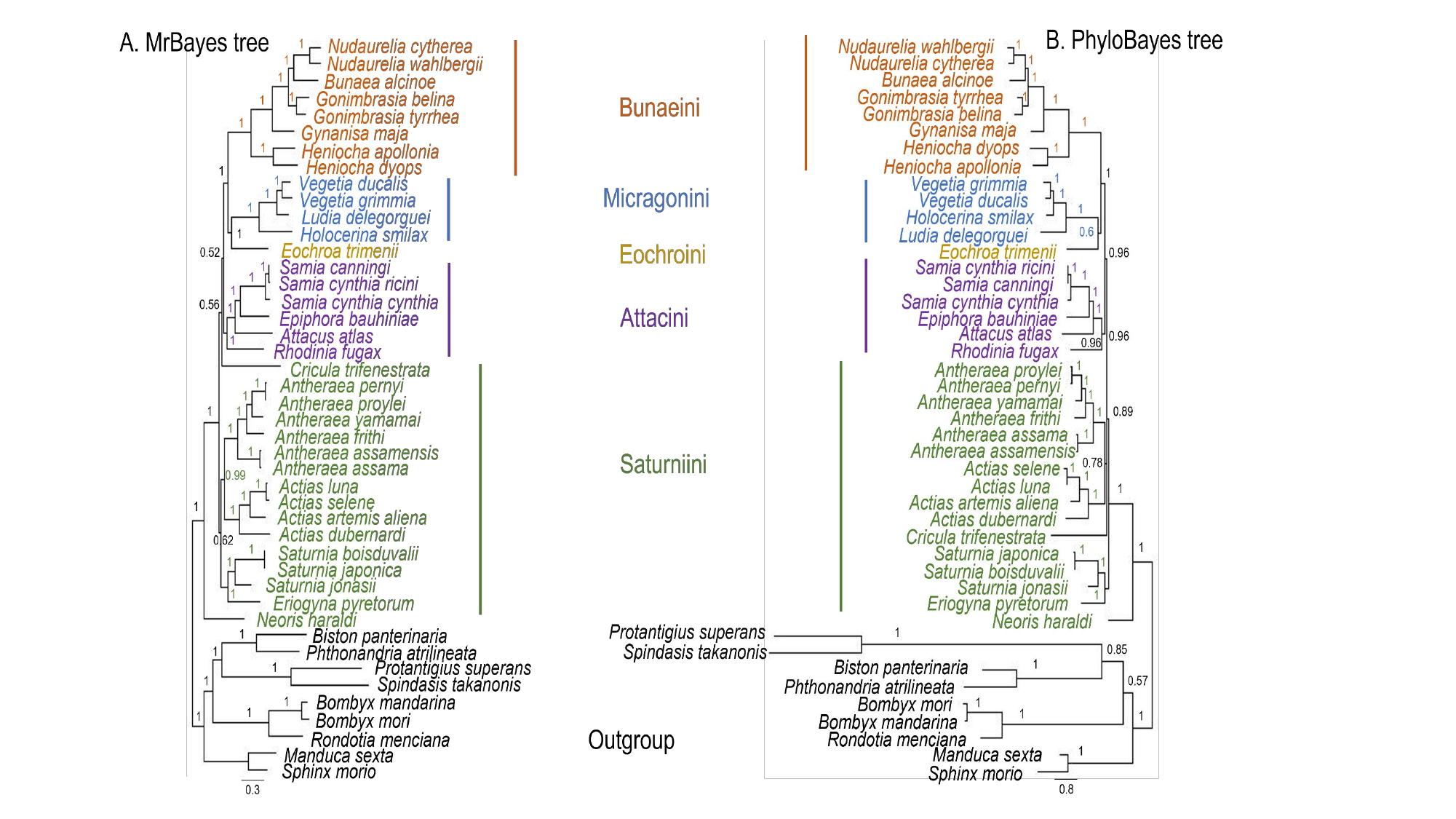

Supplement: Supplemental Information 7 — Bayesian inference trees of Saturniidae species based on 13 mitochondrial protein-coding genes. Nodal support is given as Bayesian posterior probabilities. [file peerj-10-13275-s007.pptx]

## Slide 1
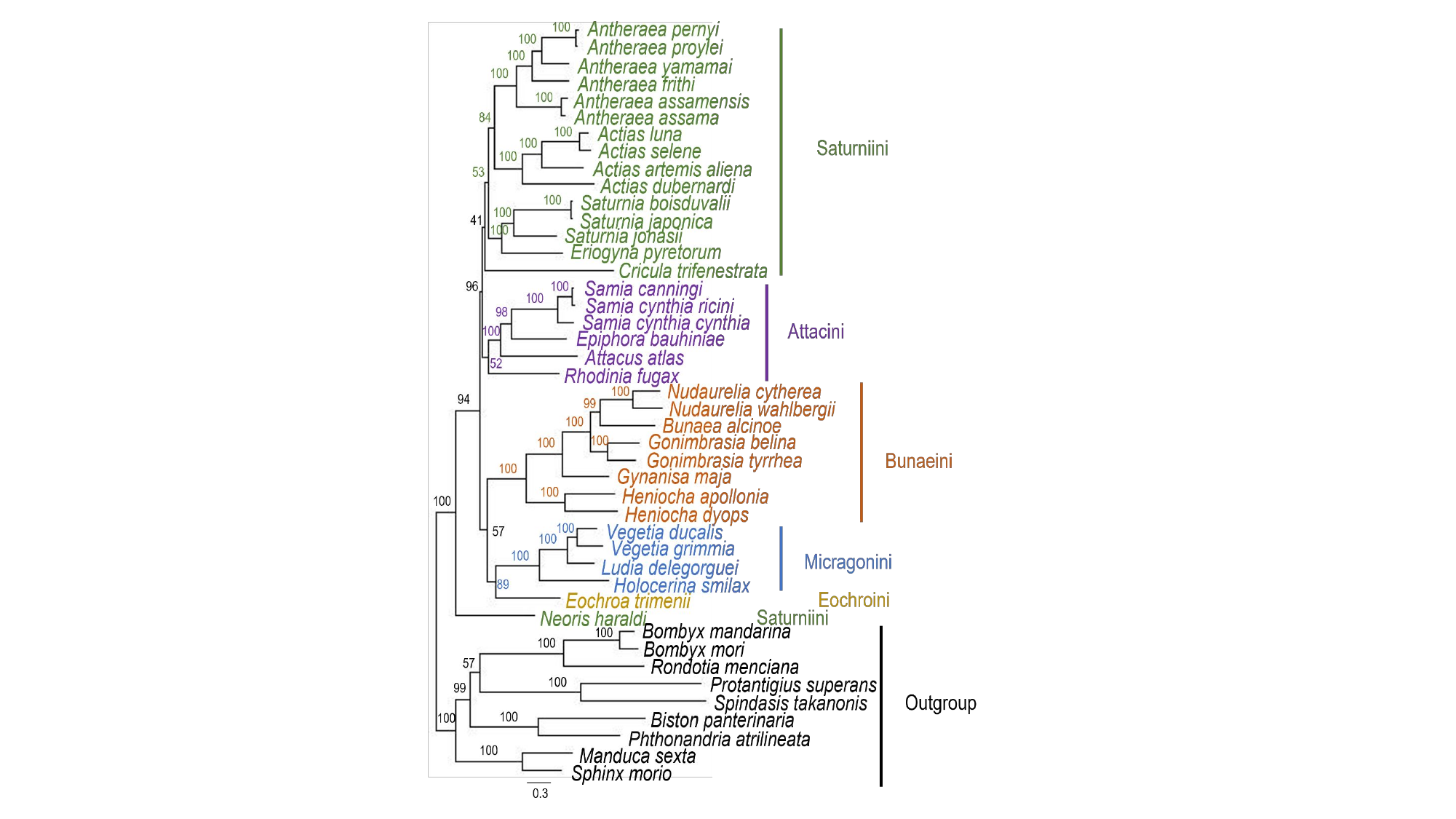

Supplement: Supplemental Information 8 — Maximum likelihood tree of Saturniidae species based on 13 mitochondrial protein-coding genes. Nodal support was based on 1,000 bootstrap replicates. [file peerj-10-13275-s008.pptx]
